# Demobilisation and Venereal Diseases

**Published:** 1919-10-18

**Authors:** 


### DEMOBILISATION AND VENEREAL DISEASES.

The question of venereal disease is not a new one. It has troubled Europe for centuries and America certainly since that great continent was discovered, even if the tale be not true that Columbus and his crews brought syphilis to Spain. After great wars, so far as the statistics of the past may be trusted, there has usually or always been a great increase of infection brought into their native lands by returning soldiers. It is expected that this war will be no exception and it is claimed that already, before the war is ended, there is in Great Britain a great increase in the number of infected persons of both sexes. The statistics have not been compiled with exactness or with system. In fact the statement seems to be an expression of opinion, not a result of careful research and compilation of figures. Nor is it very certain that there exist sufficiently exact statistics of the state of affairs before the war to make comparison valuable. There is, however, no doubt that a very large number of persons are syphilitic.

### Canadian Figures.

Colonel J. G. Adami, F.R.S., states that from eight to twelve per cent. of the civil population of Canada give a possitive Wassermann reaction and two per cent. of the returning soldiers. The figures are for Canada. As soldiers actively infectious are not returned to Canada the real percentage amongst them must be much higher than two. Eight to twelve per cent. seems a very high figure, and to it must be added a large number of persons who have gonorrhœa but have not got syphilis in order to obtain the total of persons suffering from venereal disease. When it is remembered that a nation is supposed to be able to put in the field about ten per cent. of its gross population and that it is chiefly persons of the military age, whether male or female, who suffer from venereal disease, the extent of the calamity becomes appalling. Is the figure as high for Great Britain?

Colonel Adami thinks that the returning soldiers will get more venereal disease from the civil population than they will give it. His remedy is, or rather the best method of preventing the disease that he can suggest is, first, lectures, persuasion and advice of a nature to prevent young men from seeking loose women, or from succumbing to temptation when they meet them, and secondly early prophylactic treatment both before and after copulation. He pleads earnestly for both times of prophylaxis and exposes the fallacy of the contention that whilst it is right to take measures to prevent the consequences of impure connections after the act, or to make use of any and every known remedy for the consequences if consequences ensue, it is wrong to prepare for the act by prophylactic inunction or other method of avoiding infection.

### The Fringe of the Question.

But this is only the fringe of the question. The fact will remain that there will be a vast population of infectious persons in all countries of the world if anything approaching the Canadian figure is to obtain elsewhere. It is certain that the British public will not consent to measures which might be effective. They would include compulsory notification, and compulsory segregation till it was reasonably certain from careful examination that the infectious period was passed. Merely to state such measures and to set beside them a statement of the number of persons to whom it is said they would have to apply and a calculation of the average time of treatment of cases is enough to ensure their rejection as impracticable. The problem seems to be insoluble, and it seems at first as if the human race must therefore perish of the results of its sexual instincts or evolve a race immune to these diseases.

There is a grain of comfort to be found in contemplation of the past. There have been wars before, and after the wars an increase of venereal disease; yet the human race continues to put forth offspring not infected—if it were not so there would be no need for alarm about infection—and not only that, it puts forth men and women in quantity as fine and as strong as any that went before them. It is a terrible thing to see a fine man wrecked, to know that the hopes of children of a might-have-been good mother are blighted for ever, to see the misery and poverty and horribleness that these diseases bring to many of their victims; but somehow it seems as if nature had ways by which to purge the kind, to work out the sickness from it and to secure that a portion should survive free of the taint and able to keep high the standard of the best of the race. Research may reveal the process, for some such process surely there must be. Probably even the virus of syphilis is often less strong than vis medicatrix naturæ.

